# How Age and Linguistic Competence Affect Memory for Heard Information

**DOI:** 10.3389/fpsyg.2016.00618

**Published:** 2016-05-09

**Authors:** Bruce A. Schneider, Meital Avivi-Reich, Caterina Leung, Antje Heinrich

**Affiliations:** ^1^Human Communication Laboratory, Psychology, University of Toronto MississaugaMississauga, ON, Canada; ^2^Interdisciplinary Center Herzliya Israel, PsychologyHerzliya, Israel; ^3^Medical Research Council Institute of Hearing ResearchNottingham, UK

**Keywords:** second language speakers, auditory memory, context, age, spoken word recognition, spoken language comprehension

## Abstract

The short-term memory performance of a group of younger adults, for whom English was a second language (young EL2 listeners), was compared to that of younger and older adults for whom English was their first language (EL1 listeners). To-be-remembered words were presented in noise and in quiet. When presented in noise, the listening situation was adjusted to ensure that the likelihood of recognizing the individual words was comparable for all groups. Previous studies which used the same paradigm found memory performance of older EL1 adults on this paired-associate task to be poorer than that of their younger EL1 counterparts both in quiet and in a background of babble. The purpose of the present study was to investigate whether the less well-established semantic and linguistic skills of EL2 listeners would also lead to memory deficits even after equating for word recognition as was done for the younger and older EL1 listeners. No significant differences in memory performance were found between young EL1 and EL2 listeners after equating for word recognition, indicating that the EL2 listeners' poorer semantic and linguistic skills had little effect on their ability to memorize and recall paired associates. This result is consistent with the hypothesis that age-related declines in memory are primarily due to age-related declines in higher-order processes supporting stream segregation and episodic memory. Such declines are likely to increase the load on higher-order (possibly limited) cognitive processes supporting memory. The problems that these results pose for the comprehension of spoken language in these three groups are discussed.

## Introduction

A listener's ability to comprehend a lecture, or a multi-talker conversation, is usually measured by having the listener answer questions about the discourse they heard. Clearly, the ability to store the information contained in the lecture or conversation for later recall is one of many abilities that are required in order to perform well on this test of speech comprehension. Consequently, we would expect speech comprehension in individuals who were less proficient than others in either storing or retrieving the heard information, to be poorer than in those individuals whose memory is unimpaired. All other things being equal, those who have good memory are likely to outperform those with poorer memory. Older adults are one group that suffer from declines in memory processes (Ohta and Naveh-Benjamin, 2012; Morris and Logie, 2015). Second language listeners may be another (Olsthoorn et al., 2014), although the evidence here is less clear and may depend on the particular memory task (Schroeder and Marian, 2014).

However, memory is not the only determinant of performance in a conversational situation, especially when there are competing sound sources. Speech recognition is also vital. As a consequence of poorer perceptual skills, people who find it difficult to hear the individual words in connected discourse will most likely find it difficult to extract the information in an utterance, integrate the extracted information with past knowledge, and store it in memory for later recall. This will result in less efficient recall of what they have heard compared to those who were experiencing fewer difficulties with respect to word recognition. Hence, difficulties in remembering heard information could result from compromised speech perception, reduced memory ability, or both. One way to differentiate between these alternatives is to equate groups of listeners with respect to word recognition accuracy. We know that under identical listening conditions, young native English listeners have better word recognition than either: (a) older native English listeners, or (b) young adults for whom English is a second language. If recall differences among these groups primarily reflect group differences in word recognition, equating these groups for word recognition should substantially reduce group differences in recall. However, if older adults, and possibly younger adults listening in their second language, also experience genuine memory difficulties in noisy situations, group difference in recall should remain after equating all individuals for their ability to recognize individual words.

In the present study we compare the ability of three groups of listeners to remember heard material after equating for differences in word recognition: young adults listening to English words in their first or native language (young EL1 listeners), older adults listening to English words in their native language (older EL1 listeners), and young adults listening to words in their second (non-native) language (young EL2 listeners). We had the following predictions. If linguistic competence affects memory, we would expect poorer performance in the young EL2 listeners than in the young EL1 listeners even after equating for word recognition. Alternatively, if linguistic competence does not affect memory but age does, we would predict, after equating all individuals with respect to word recognition, that memory for heard words should be equivalent in the two younger groups (young EL1 and young EL2 listeners), and poorer in older EL1 listeners.

### Controlling for word recognition

One can control for individual differences in the ability to recognize individual words masked by a competing sound (such as a babble of voices) by adjusting the listening situation. This can be done in listening situations that offer little, if any, contextual support for word recognition of masked words using a two-stage process. First, determine the threshold for detecting the presence of the masker (in these experiments, a babble of voices). Then present the target voice at a fixed level above each individual's threshold for detecting babble. Second, find the Signal-to-Noise Ratio (SNR) at which an individual is able, 50% of the time, to repeat accurately the last word in low-context sentences such as “Jane was thinking about coffee,” when such sentences are masked by noise (in this case, a babble of voices). The low-predictability sentences of the Revised Speech Perception in Noise Test (R-SPIN, Bilger et al., 1984) can be used to determine this SNR, because the context preceding the last word of the sentence provides only minimal clues as to the identity of the last word. Knowledge of each individual's babble threshold, and his or her 50% threshold for sentence final word recognition, can then be used to individually adjust the listening situation so that word recognition in the absence of contextual support (the probability of correctly identifying the word being spoken) is comparable for all individuals regardless of their hearing status, or of their age.

### Memory for words presented in background noise when listening in one's second language

Some studies have shown episodic memory in a word recall task to be poorer in young EL2 than in young EL1 listeners after listening to a series of words (e.g., Fernandes et al., 2007). This could be due to a number of factors related to their linguistic ability. For instance, we might expect the lexicon to be less fully developed in one's second language than in one's first language (Bialystok et al., 2010; Bialystok and Luk, 2012). Second, the target speech stream might be expected to initiate activity in the individual's first language lexicon as well as in the second-language lexicon (Schroeder and Marian, 2014). Deficiencies in one's second language lexicon, coupled with dual activation of both the first- and second-language lexicons could make it more difficult to encode the heard words into long-term memory. A third reason is poorer discriminability for certain phonemic contrasts, especially when noise is present (e.g., Garcia Lecumberri and Cooke, 2006). Because Fernandes et al. (2007) took no steps to equate individuals with respect to word recognition other than presenting the words in quiet, we do not know whether memory in young EL2 listeners would continue to be poorer than in young EL1 listeners once the listening situation is individually adjusted in all participants to achieve equivalent levels of word recognition for to-be-encoded words. Hence, if reduced perceptual accuracy and discriminability play a critical role for young EL2 listeners' speech perception and memory abilities, equating their perceptual accuracy to that of their first language counterparts (young EL1) should minimize memory differences between the two groups. If equating for perceptual differences does not equate for memory differences, then differences in other linguistic abilities such as size and activation of the mental lexicon must play an important role. If this were to be the case, young EL2 listeners' memory performance could resemble that of older EL1 listeners even after equating for word recognition (Murphy et al., 2000; Heinrich and Schneider, 2011).

If a poorer memory for EL2 listeners is found even after adjusting for word recognition, an exploration of how memory is affected by the parameters of the competing noise could help us to identify the reasons for poorer memory in one's second language than in one's first language. An examination of the similarities and differences in the patterns of memory deficits in young EL2 and older EL1 listeners could potentially help us identify the nature and comparability of the memory deficits in both groups. For these reasons, in this study we compared memory performance in a paired-associate memory paradigm for heard words identical to that previously used to obtain data from young and old EL1 listeners.

Presenting all stimuli in a background noise makes it easier to equate for perceptual differences between individuals. However, it is not only the presence or absence of noise that has previously been found to affect memory but also the temporal relationship of the background noise to the word presentation (Heinrich and Schneider, 2011). Therefore, we investigated recall in two different background noise conditions: continuous noise, and noise only present during word presentation. In addition we also collected data for a quiet baseline condition. In a previous study we found that for young EL1 listeners, only the presence of continuous noise led to a reduction in memory compared to the quiet condition whereas both kinds of noise led to memory deficits for older EL1 listeners (Heinrich and Schneider, 2011). If the same pattern as the one found in older EL1 listeners occurred for young EL2 listeners, we might conclude that the underlying processes governing recall in noise reflect similar deficiencies in the processes supporting paired-associate memory. Conversely, if after equating for word recognition, paired-associate memory for heard words was found to be equivalent in young EL1 and EL2 listeners, we could conclude that the language proficiency of listeners did not affect their memory for heard words. Such a finding would be consistent with the hypothesis that the reasons why older EL1 listeners perform poorer than young EL1 listeners even after equating for word recognition, is due primarily to age-related changes in higher-order cognitive processes supporting episodic memory.

**Figure 1:**
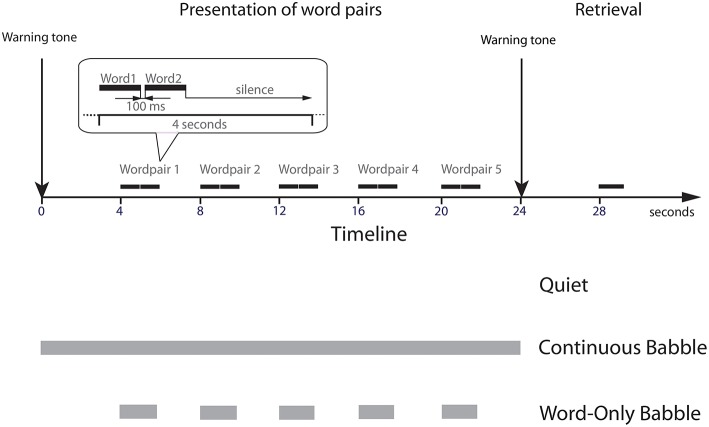
**An illustration of the three stimulus conditions tested (Quiet, Continuous Babble and Word-Only Babble)**. The beginning of a trial was signaled by a warning tone, followed 4 s later by the first word pair. Subsequent word pairs were spaced 4 s apart with 100 ms separating the two words in a pair. A warning tone followed 4 s after word pair five. The first word of one of the word pairs was presented in quiet 4 s later. In the Continuous Babble condition, the babble was played continuously between the warning tones. For Word-Only Babble, the babble began and ended with the word pair.

Hence, in the present experiment we compared memory for heard paired associates obtained in previous experiments for younger and older adults listening in their first language (young EL1 listeners, older EL1 listeners, Murphy et al., 2000; Heinrich and Schneider, 2011) to data collected here on young EL2 listeners. In all three groups, the average sound pressure at which the word pairs were presented and the SNR at which they were presented in the background babble were adjusted to produce equivalent levels of word recognition in the absence of contextual support in all three groups. The paired associates were presented under three different masking conditions: (1) no masking (Quiet); (2) Continuous masking by a 12-talker babble of voices; and (3) Word-Only masking where the onset and offset of the masker was contemporaneous with the onset and offset of the word pair (see Figure 1). Four seconds after the last paired associate was presented, a warning tone was sounded. Four seconds later, the first word of one of the paired associates was presented in quiet. These three masking conditions were chosen because the pattern of results for young EL1 listeners for these three maskers differed substantially from the pattern of results on the same three maskers in older EL1 listeners. Hence we felt that an exploration of how young EL2 listeners might perform under these three masking conditions would allow us to identify (1) the ways in which memory might differ in young EL1 and young EL2 listeners; and (2) shed some light on the nature of the perceptual and/or cognitive factors that might be responsible for the memory deficits in older adults.

## Methods

### Materials and methods

#### Young EL2 participants

**Figure 2:**
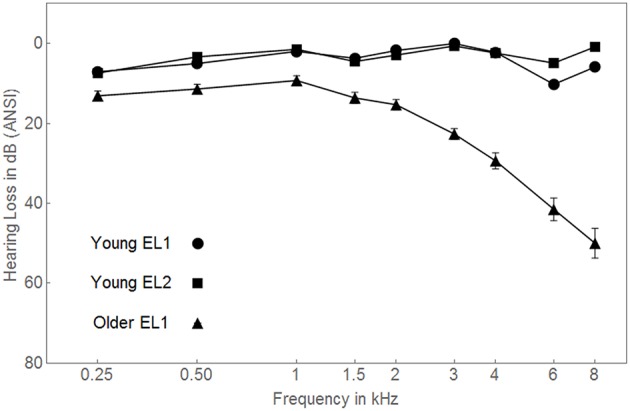
**Average audiograms for the three groups of participants are shown for the left ear**. ANSI, American National Standards Institute. Standard error bars are shown.

A total of 90 EL2 undergraduate students at the University of Toronto (30 students in each of the three conditions) were paid $10 per hour for their participation. All participants first became immersed in an English speaking environment after the age of 7 years, and were not extensively exposed to English prior to that. Details concerning their age, gender, age of arrival in an English-speaking country, years of education, Mill Hill vocabulary scores, and Nelson-Danny reading comprehension scores are presented in Table 1 separately for each of the three testing conditions. All participants were required to have clinically normal hearing. Pure-tone air-conduction thresholds were measured at nine frequencies (0.25–8 kHz) for both ears using an Interacoustics Model AC5 audiometer (Interacoustic, Assens, Denmark). All participants were required to have pure tone air-conduction thresholds of 15 dB HL or lower, between 0.25 and 8 kHz in both ears. Participants with a threshold of 20 dB HL at a single frequency were not excluded from the study. Participants who demonstrated unbalanced hearing (more than a 15 dB difference between ears at one or more frequencies) were excluded from participation. The average audiograms for the 90 EL2 participants are shown for the left ear only in Figure 2 (circles). During each participant's first experimental session we administrated audiometric thresholds, the Nelson-Denny reading comprehension test (Brown et al., 1981) and the Mill Hill test of vocabulary knowledge (Raven, 1965). The memory task, along with the babble detection thresholds and the low-context R-SPIN thresholds were administered over the next experimental session. All experimental procedures were approved by the Research Ethics Board of the University of Toronto.

**Table 1:** **Participant parameters for the young EL2 and EL1 listeners under the three conditions tested along with those of the older EL1 listeners**.

	**Condition**	***N***	**Age range**	**Age**		**Gender**		**Immersion(Age)**		**Education**		**Vocab. knowledge**		**Reading comp**.	
				**M**	***SD***	**Male**	**Female**	**M**	***SD***	**M**	***SD***	**M**	***SD***	**M**	***SD***
Younger EL2	Continous	30	18–24	20.07	1.60	5	25	13.23	4.49	14.1	1.12	9.37	3.86	18.67	6.69
	Word-Only	30	18–24	20.30	1.74	10	20	14.73	3.85	14.2	1.65	9.93	2.39	21.00	5.68
	Quiet	30	18–25	20.37	2.13	10	20	13.97	4.91	14.2	1.94	8.77	3.04	18.80	5.38
Younger EL1	Continuous^**^	15	19–25	21.87	1.88	3	12			17.00	2.00	13.20	3.14		
	Word-Only^***^	16	18–23	19.69	1.35	4	12			15.06	1.88	13.38	1.63		
	Quiet^*^	15	19–25	21.27	2.15	7	8			15.53	2.33	13.80	0.45		
Older EL1	Continuous^**^	15	66–88	72.60	6.07	9	6			13.73	3.01	16.00	2.83		
	Word-Only^***^	17	65–84	72.41	6.47	4	13			13.59	2.69	14.29	2.02		
	Quiet^*^	15	65–79	71.33	4.40	7	8			14.50	2.28	16.87	1.46		

*Reading comprehension scores were not available for the participants in Murphy et al. (2000) as well as in Heinrich and Schneider (2011). Age of immersion is not relevant for the younger and older EL1 listeners*.

* *Data was taken from Murphy et al. (2000), Experiment 2*.

** *Data was taken from Murphy et al. (2000), Experiment 3*.

*** *Data was taken from Heinrich and Schneider (2011), Experiment 2*.

#### Younger and older EL1 participants

The data for the younger and older EL1 listeners in the Quiet condition were taken from Experiment 2 of Murphy et al. (2000). The data for the younger and older EL1 listeners in the Continuous Babble condition were taken from Experiment 3 of Murphy et al. Finally, the data for the younger and older EL1 listeners in the Word-Only Babble condition were taken from Heinrich and Schneider (2011). The younger adults were also University of Toronto undergraduates, and were tested under the same conditions as the young EL2 listeners in the present experiment. The older EL1 listeners were volunteers from the Mississauga community, and tested under the same conditions as the young EL2 listeners in the present experiment. Their numbers, ages, years of education, and vocabulary scores are reproduced in Table 1. All EL1 listeners were immersed in an English-speaking environment before the age of 5. Reading comprehension scores were not available for these participants. The left-ear Babble and R-SPIN thresholds appear in Table 2.

**Table 2:** **Babble and SPIN thresholds in the left ear for each of the conditions and groups**.

**Group**	**Condition**	**Left ear thresholds**			
		**Babble**		**R-SPIN**	
		**dB SPL**	***SD***	**SNR in dB**	***SD***
Younger EL2	Continuous	15.92	2.35	4.90	3.54
	Word-Only	15.09	2.95	5.76	3.00
	Quiet	15.29	3.25	No babble noise	
Younger EL1	Continuous^**^	15.32	3.23	−0.12	1.26
	Word-Only^***^	15.30	2.92	0.44	2.25
	Quiet^*^	17.81	8.65	No babble noise	
Older EL1	Continuous^**^	23.95	7.13	3.09	1.96
	Word-Only^***^	25.56	8.41	4.24	3.47
	Quiet^*^	23.91	5.89	No babble noise	

* *Data was taken from Murphy et al. (2000), Experiment 2*.

** *Data was taken from Murphy et al. (2000), Experiment 3*.

*** *Data was taken from Heinrich and Schneider (2011), Experiment 2*.

#### General methods

The stimuli, apparatus, and testing protocols were taken from Murphy et al. (2000), Heinrich et al. (2008) and Heinrich and Schneider (2011). Hence any differences between the present results and those previously found in these studies cannot be attributed to differences in any of these factors.

##### Apparatus and stimuli

The word pairs, which were the same as those in Murphy et al. (2000), consisted of 400 two-syllable common nouns with a frequency of more than 1 per million (Kucera and Francis, 1967). The individual words, spoken by a female speaker, were digitally recorded at a sampling rate of 20 kHz and had similar root-mean-square (RMS) values. The word pairs were delivered through a 16-bit digital-to-analog converter (TDT DD2) followed by a 10-kHz low-pass filter to the left ear only. All testing took place in a double-walled sound-attenuating chamber using headphones.

##### Procedure babble threshold

The words were presented at a level that was individually set to 50 dB above the listener's babble threshold. Adjusting presentation level individually was important because older adults' babble thresholds are considerably higher than those of younger adults. If an identical presentation level for both age groups had been used, the stimuli would have been presented too close to the old listeners' threshold, which could have an adverse effect of word recognition. To individually adjust presentation level, the 12-talker babble materials used in these experiments were taken from the Revised Speech Perception in Noise (R-SPIN) test (Bilger et al., 1984). Thresholds for the detection of babble when presented to the left ear only were determined for each individual allocated to one of the two noise conditions (Word-Only and Continuous Babble). We used a two-interval, two-alternative forced-choice paradigm with an adaptive three down one up procedure (Levitt, 1971) to determine the babble threshold corresponding to the 79% point on the psychometric function. In this procedure, a 1.5 s babble segment was randomly presented in one of two intervals which were separated by a 1.5-s silent period. Two lights on the button box indicated the occurrence of each interval, and the listener's task was to identify the interval containing the babble segment by pressing the corresponding button. Immediate feedback was provided after each press by flashing the LED corresponding to the interval in which the babble segment occurred (for more details see Heinrich et al., 2008; Heinrich and Schneider, 2011). The starting intensity was 50 dB SPL. The intensity of the babble was reduced after three correct responses in a row and increased after a single incorrect response. The session was terminated after 12 reversals. The babble threshold was defined as the average SPL on the last eight reversals. Babble thresholds for the left ear (all stimuli were presented to the left ear only) are shown in Table 2.

##### Individually adjusting the signal-to-noise ratio

Following Murphy et al. (2000), and Heinrich and Schneider (2011), the low-context sentences from the R-SPIN test (Bilger et al., 1984) were used to determine the SNR for each individual that resulted in 50% correct identification of the last word in these sentences. Participants were asked to immediately repeat the last word of individual sentences presented to them in a multi-talker babble background. Each participant listened to at least two R-SPIN lists played to his or her left ear, at SNRs that were chosen to bracket the 50% final words' intelligibility point in low-context sentences (e.g., “Jane was thinking about coffee”). The SNR corresponding to the 50% point was then estimated by linear interpolation and is shown in Table 2 for all groups. The SNR used in the memory task, was set at the individual SNR value corresponding to 50% correct identification minus 7 dB, which was shown by Murphy et al. (2000) to result in approximately 91% correct word identification when the words used in the memory experiments were presented in babble. Consider the following example in which the listening situation is individually adjusted for two individuals, one younger, and the other older, to produce equal word recognition in the absence of contextual support. Suppose the thresholds for detecting a babble of voices are 10 and 18 dB SPL for the younger and older adult, respectively. To equate for individual differences in babble threshold, the target sentence is presented at, say, 50 dB above each individual's babble threshold (at 60 and 68 dB SPL, for the younger and older individuals, respectively). Now suppose we want to set the nominal SNR to −7 dB. Suppose the low-predictability R-SPIN threshold for the younger individual is −1 dB, whereas it is 4 dB for the older individual, a 5 dB difference. The babble level for the younger listener would be set to 60 + 7 dB + 1 = 68 dB SPL, for an SNR of −8 dB. The babble level for the older individual would be set to 68 + 7−4 = 71 dB SPL producing an SNR of −3 dB. Note that the SNR for the older individual is 5 dB higher than that of the younger individual, which is equal to the difference in the R-SPIN thresholds for low-predictability sentences.

Previous studies have shown that the psychometric functions relating percent correct word recognition to SNR have equivalent slopes for younger and older adults (Ben-David et al., 2012), and that the slopes for younger adults do not differ substantially for EL1 and EL2s (Zhang et al., 2014). Hence, once the SNRs are individually adjusted for 50% word recognition, changes away from the adjusted value should produce equivalent performance across age and language experience in the absence of contextual support. Thus, by individually adjusting the SNRs we equated for individual differences in word recognition in noise when there is no assisting context. Table 2 presents the average SNR for 50% intelligibility of a low-context sentence under each of the two babble conditions.

##### Word recall

As in the previous studies of this series, participants listened to words that were randomly arranged in 40 lists containing five word pairs following the paradigm by Madigan and McCabe (1971). Four seconds after a short warning tone, the first word pair was presented with a silent period of 100 ms between the words. The inter-stimulus-interval between successive word pairs was also 4 s. Another 4 s after the presentation of the last word pair of the list, another short warning indicated the beginning of the recall phase (for more details see Heinrich and Schneider, 2011). Participants were cued with the first word from one of the five previously presented word pairs and were asked to recall the second word which was presented as part of the same pair. Only one pair from each list was cued; no time limit was placed on recall, and participants were encouraged to guess. The serial positions refer to the order in which the word pairs were presented in each trial; the first serial position refers to the first word pair. The serial position of each word pair within the five-word-pair list was tested eight times within a session. The list order was identical for all participants, and the order in which the serial positions were tested was independently and randomly determined for each participant. No feedback was provided. Participants were instructed to take a break after the presentation of the first 20 lists.

The word pairs were presented under three different masking conditions: (1) no masking (Quiet); (2) Continuous masking by a 12-talker babble of voices; and (3) Word-Only masking where the onset and offset of the masker was contemporaneous with the onset and offset of each of the word pairs (see Figure 1). Three independent groups of participants were tested in each masker condition.

## Results

### Babble detection thresholds, R-SPIN word recognition thresholds, and mill hill vocabulary

Babble thresholds, R-SPIN thresholds, and Mill Hill vocabulary scores were obtained for all of the participants in both Continuous Babble and Word-Only Babble in all three groups (see Table 2). For babble and R-SPIN thresholds, as well as for Mill Hill Vocabulary scores, we might expect to find differences among the three groups but not among masking conditions nor any interaction between masker type and group. A Between-Subjects ANOVA with three Groups (young EL1 listeners, young EL2 listeners, and old EL1 listeners) and two Masker Types (Continuous vs. Word-Only) indicated that babble thresholds differed across groups [*F*_(2, 117)_ = 32.357, *p* < 0.001, η_*p*_^2^ = 0.356] but not across Masker Type [*F*_(1, 117)_ < 1]. In addition the Group × Masker Type interaction was not significant [F_(2, 117)_ < 1]. *Post-hoc* LSD tests found that both younger groups had lower babble thresholds than the older group (*p* < 0.001 in both instances) with no difference between the younger EL1 and EL2 groups (*p* = 0.859). A Between-Subjects ANOVA on the R-SPIN thresholds found that R-SPIN thresholds differed across groups [*F*_(2, 117_) = 33.688, *p* < 0.001, η_*p*_^2^ = 0.365] with no significant difference between the two Masker Conditions [*F*_(1, 117)_ = 2.127, *p* = 0.147] and no significant interaction between the two factors [*F*_(2, 117)_ < 1]. *Post-hoc* LSD tests indicated that R-SPIN thresholds were order from lowest to highest as young EL1, old EL1, young EL2 with young EL1 listeners having significantly lower R-SPIN thresholds than the other two groups (*p* < 0.001 for both comparisons), and older EL1 listeners having significantly lower thresholds than young EL2 listeners (*p* = 0.009).

A Between-Subjects ANOVA with three Groups (young EL1 listeners, young EL2 listeners, and old EL1 listeners) and two Masker Types (Continuous vs. Word-Only) indicated that Mill Hill vocabulary scores differed across groups [*F*_(2, 117)_ = 42.801, *p* < 0.001, η_*p*_^2^ = 0.423] but not across Masker Type [*F*_(1, 117)_ < 1]. In addition the Group × Masker Type interaction was not significant [*F*_(2, 117)_ = 1.703, *p* = 0.187]. *Post-hoc* LSD tests found that Mill Hill vocabulary scores were ordered from lowest to highest as young EL2, young EL1, and old EL1, with young EL2 listeners having lower Mill Hill vocabulary scores than did both of the other two groups listeners (*p* < 0.001 for both comparisons), and young EL1 listeners having lower vocabulary scores than older EL2 listeners (*p* = 0.014). There were no main effects of Masker Type on babble thresholds, R-SPIN thresholds, and Mill Hill vocabulary scores, nor any evidence of interaction between Masker Type and Groups for these three variables. This indicates that any effects of Masker Type on memory performance cannot be attributed to the use of different participants for the three different types of maskers.

### Paired-associate memory: young EL2 vs. young EL1 participants

In these experiments, the levels at which the words were presented, and the SNR at which they were presented, were adjusted to achieve the same level of word recognition in all participants. To determine whether the linguistic status of the listener affected their ability to recall the second word in a pair when prompted with the first word of a pair, we conducted a three factor ANOVA on the percentage of words correctly recalled in each serial position for young EL1 listeners (taken from Murphy et al., 2000) and the EL2 listeners. In this analysis the serial position of the word was a within-subject factor. The two other factors, Type of Masker (none, Continuous Babble, Word-Only Babble), and language status were between subjects factors. This analysis revealed a main effect of Serial Position [*F*_(4, 520)_ = 137.543, *p* < 0.001, η_*p*_^2^ = 0.514], a main effect of Masker Type [*F*_(2, 130)_ = 6.419, *p* = 0.002, η_*p*_^2^ = 0.090], and an interaction between Serial Position and Masker Type [*F*_(8, 520)_ = 3.617, *p* < 0.001, η_*p*_^2^ = 0.053]. *Post-hoc* LSD tests indicated that performance in quiet was better than in Continuous Babble (*p* = 0.001), and better than in Word-Only Babble (*p* = 0.042), but that there was no overall difference between Continuous Babble and Word-Only Babble (*p* = 0.176). Neither the main effect of Language Status, nor any of the interactions involving Language Status reached statistical significance: Language Status [*F*_(1, 130)_ < 1]; Serial Position × Language Status [*F*_(8, 520)_ = 1.318, *p* = 0.262]; Serial Position × Language Status × Masker Type [*F*_(8, 520)_ < 1]. Hence, there is no evidence that recall is affected by language status as long as the listening situation is adjusted to achieve equal levels of word recognition in both young EL1 and young EL2 listeners. Because we did not find any effect of language status between young EL1 and EL2 participants, we aggregated the data from both groups in subsequent analyses.

**Figure 3:**
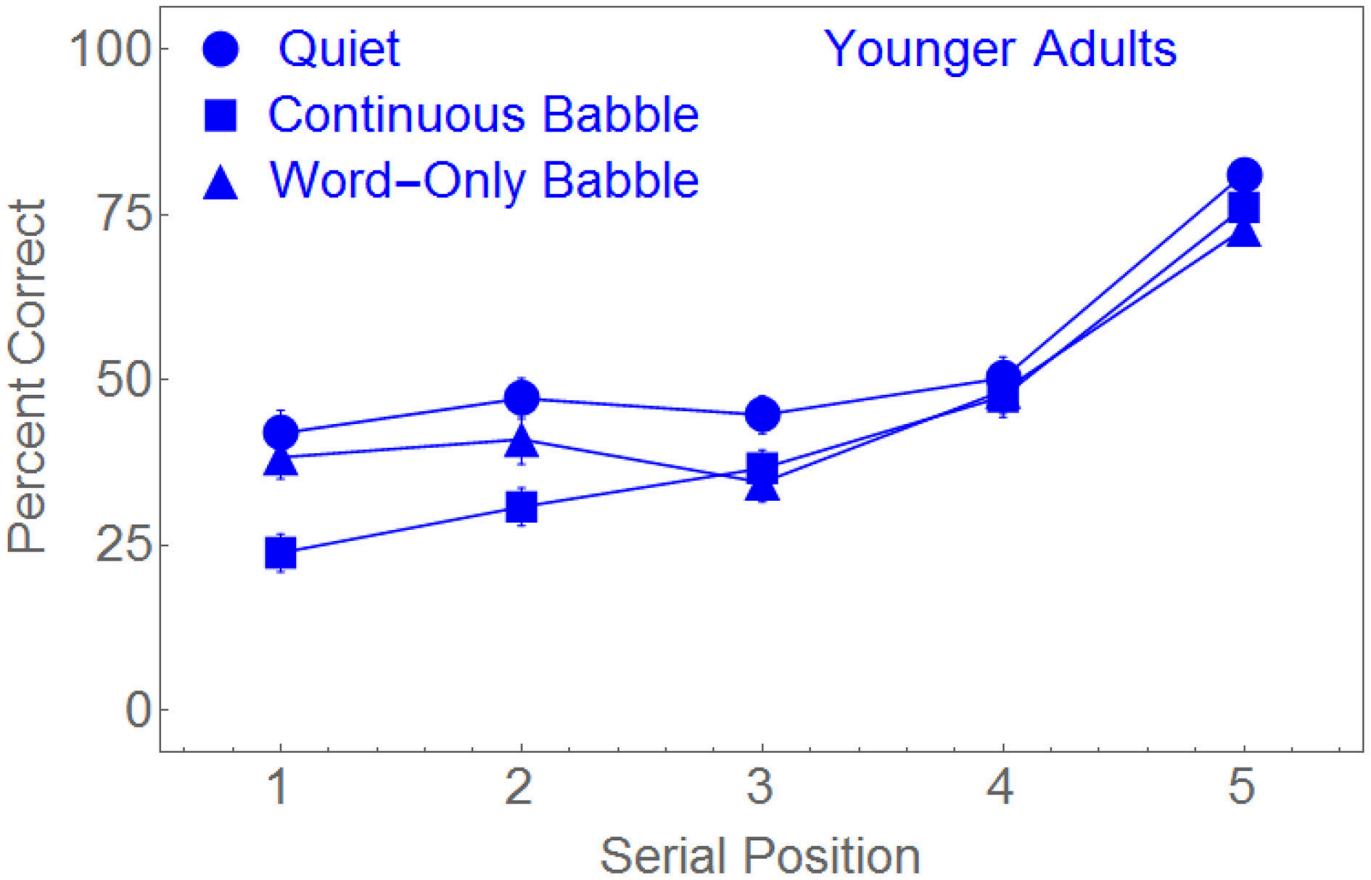
**Percent correct word identification as a function of the serial position of the word pair for the younger adults in three different masking conditions**. Standard error bars are shown.

Figure 3 plots the percentage of words correctly recalled as a function of serial position for each of the masking conditions. Figure 3 suggests that the performance of young adults was roughly identical under all masker conditions for serial positions 4 and 5. When the word pairs were presented in Quiet or in Word-Only Babble, performance appears to be equivalent for serial positions 1, 2, and 3. However, when a Continuous Babble was used as a masker, performance appeared to be significantly lower in positions 1 and 2 than in the other two masker conditions. To confirm that the difference in performance among the three maskers in the early serial positions was responsible for the Serial Position × Masker Type interaction, we conducted three additional ANOVAs. In each of these ANOVAs, Serial Position was a within-subject factor. In the first ANOVA the second factor (Masker Type) contained only two levels (Quiet and Continuous Babble). In the second ANOVA, the two levels of Masker Type were Quiet and Word-Only Babble. In the third ANOVA, the two levels of Masker Type were Continuous Babble and Word-Only Babble. Significance in these three ANOVAs were Bonferroni corrected. Not surprisingly, the main effect of serial position was highly significant in all three ANOVAs (*p* < 0.001, η_*p*_^2^ > 0.48). When the masker contrast was between Quiet and Word-Only Babble there was no significant main effect due to Masker Type [*F*_(1, 89)_ = 3.681, *p* > 0.2], nor was there a significant Serial Position × Masker Type interaction [*F*_(4, 356)_ < 1]. However, when the masker contrast was between Quiet and Continuous Babble, there was significant main effect of Masker Type [*F*_(1, 88)_ = 13.308, *p* < 0.01, η_*p*_^2^ = 0.131] and a significant Serial Position × Masker Type interaction [*F*_(4, 352)_ = 3.812, *p* < 0.01, η_*p*_^2^ = 0.042]. Finally, when the masker contrast was between Continuous Babble and Word-Only Babble the main effect due to Masker Type was not significant [*F*_(1, 89)_ = 1.788, *p* > 0.5] whereas the interaction between Serial Position and Masker Type was [*F*_(4, 356)_ = 5.165, *p* < 0.01, η_*p*_^2^ = 0.055]. Hence, for young adults, performance in the Word-Only Babble appears to be equivalent to performance in Quiet, with performance in Continuous Babble being worse than in the other two masking conditions for serial positions 1 and 2.

### Paired-associate memory: younger adults vs. older adults

**Figure 4:**
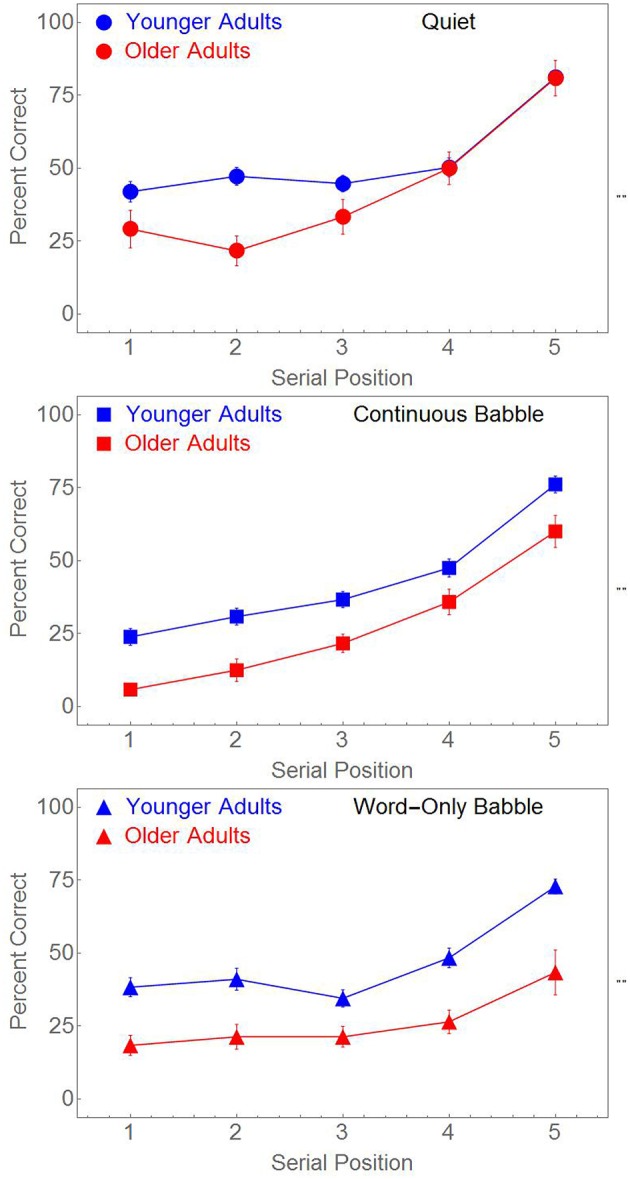
**Percent Correct word identification as a function of the serial position of the word pairs for younger and older adults**. Different masking conditions are shown in panel (as labeled). Standard error bars are shown.

Paired-associate memory in younger adults was compared to that of older adults in a 2-Age × 3 Masker Types × 5 Serial Positions ANOVA with Serial Position as a within-subject factor and Age and Masker Type as between-subjects factors. There were significant main effects of Serial Position [*F*_(4, 708)_ = 154.126, *p* < 0.001, η_*p*_^2^ = 0.465], Age [*F*_(1, 177)_ = 45.166, *p* < 0.001, η_*p*_^2^ = 0.203], and Masker Type [*F*_(2, 177)_ = 12.181, *p* < 0.001, η_*p*_^2^ = 0.121]. In addition there was a significant two-way interaction between Serial Position and Masker Type [*F*_(8, 708)_ = 4.899, *p* < 0.001, η_*p*_^2^ = 0.052], and a significant three-way interaction between Age, Serial Position, and Masker Type [*F*_(8, 708)_ = 2.294, *p* = 0.020, η_*p*_^2^ = 0.025]. These effects are readily visible in Figure 4.

Figure 4 indicates that, on average, memory is poorer in all three conditions for the older adults. The top panel indicates the presence of a Serial Position × Age interaction when the word pairs are presented in Quiet. This interaction is absent when word pairs are presented in either Continuous or Word-Only Babble. To confirm this we conducted separate ANOVAs for each of the three panels to test for an Age × Serial Position interaction. Probabilities for these three separate ANOVAs were Bonferroni corrected. The Serial Position × Age interaction was statistically significant in the top panel [*F*_(4, 232)_ = 4.456, *p* < 0.01, η_*p*_^2^ = 0.071], but not in either the middle or bottom panels of Figure 4 [*F*_(4, 232)_ < 1, and *F*_(4, 244)_ = 1.467, *p* > 0.2, respectively].

The above analysis failed to reveal any significant Age × Serial Position interaction when the paired associates were masked by either Continuous or Word-Only Babble. However, the effect of serial position appears to differ between the two types of maskers. Figure 4 suggests that for a Continuous Babble masker, performance continues to decline from serial position 3 to serial position 1 whereas there is no apparent decline from position 3 to position 1 for a Word-Only Babble masker. To confirm that the effect of Serial Position differed between the two types of maskers, we conducted a 2 Masker Type × 5 Serial Position × 2 Age Group ANOVA with Serial Position as a within-subject factor, and Masker Type and Age Group as between-subjects factors. The ANOVA revealed significant main effects of Serial Position [*F*_(4, 476)_ = 94.105, *p* < 0.001, η_*p*_^2^ = 0.442], Age Group [*F*_(1, 119)_ = 44.957, *p* < 0.001, η_*p*_^2^ = 0.274], but not of Masker Type [*F*_(1, 119)_ < 1]. Age did not interact with any of the other factors [Serial Position × Age, *F*_(4, 476)_ < 1; Masker Type × Age, *F*_(1, 119)_ < 1; and Masker Type × Serial Position × Age, *F*_(4, 476)_ < 1). However, the Serial Position × Masker Type interaction was significant [*F*_(4, 476)_ = 8.116, *p* < 0.001, η_*p*_^2^ = 0.064]. To confirm that the Serial Position × Masker Type interaction was due to a decline from serial position 3 to position 1 when the masker was Continuous Babble, and an absence of decline when the masker was Word-Only Babble, we conducted separate ANOVAs for these two Masker Types on the first three serial positions only. When the masker was continuous, there was a significant main effect for the first three serial positions [*F*_(2, 116)_ = 12.193, *p* < 0.001, η_*p*_^2^ = 0.174], with a significant linear trend [*F*_(1, 58)_ = 20.856, *p* < 0.001, η_*p*_^2^ = 0.264] but not when the masker was Word Only Babble [*F*_(2, 122)_ < 1]. The Age effect was significant for both Continuous (*p* < 0.001, η_*p*_^2^ = 0.239) and Word-Only Babble (*p* < 0.001, η_*p*_^2^ = 0.196) but there was no evidence of any Age × Serial Position interaction for either masker (*p* > 0.5 for both types of maskers). Companion analyses on serial positions 4 and 5, failed to reveal any interactions between Masker, Serial Position, and Age Group. Hence the two-way interaction between Serial Position and Masker Type is due to a decline in performance from serial position 3 to serial position 1 when the babble is continuous, whereas there is no statistically significant decline over these three positions when the word pairs are presented in Word-Only Babble.

### Relationship of vocabulary knowledge and reading comprehension to average recall in EL2 listeners

All of the EL2 listeners had their vocabulary knowledge, and reading comprehension assessed using the Mill Hill vocabulary test and the Nelson Denny Reading Comprehension test^1^. In addition, we also asked them to report on their number of years of schooling. These three measures were first centered (had the means removed) in each of three masking conditions to remove any mean differences among these conditions. We then regressed these three centered measures against the average centered percent correct score in each of these conditions according to the equation

$$\begin{array}{l} y_{i}=a_{1}*\;{years}_{i}+a_{2}*\;{MH}_{i}+a_{3}*{ND}_{i} \end{array}$$

where *years*_*i*_ refers to the number of years of education for subject *i*, *MH*_*i*_ is subject *i*'s Mill Hill vocabulary score, and *ND*_*i*_ is subject *i*'s Nelson-Denny Reading Comprehension score. In this model we were unable to reject the hypothesis that *a*_1_ = *a*_3_ = 0 [*F*_(2, 87)_ = 1.296, *p* = 0.279]. Hence this reduced the model to,

$$\begin{array}{l} y_{i}=a_{2}*\;{MH}_{i} \end{array}$$

The correlation coefficient between centered percent correct and the centered Mill Hill vocabulary score was 0.34 which was significantly different from zero (*p* = 0.001).

### Relationship of vocabulary knowledge and reading comprehension to average recall in young EL1, young EL2, and old EL1 listeners

**Figure 5:**
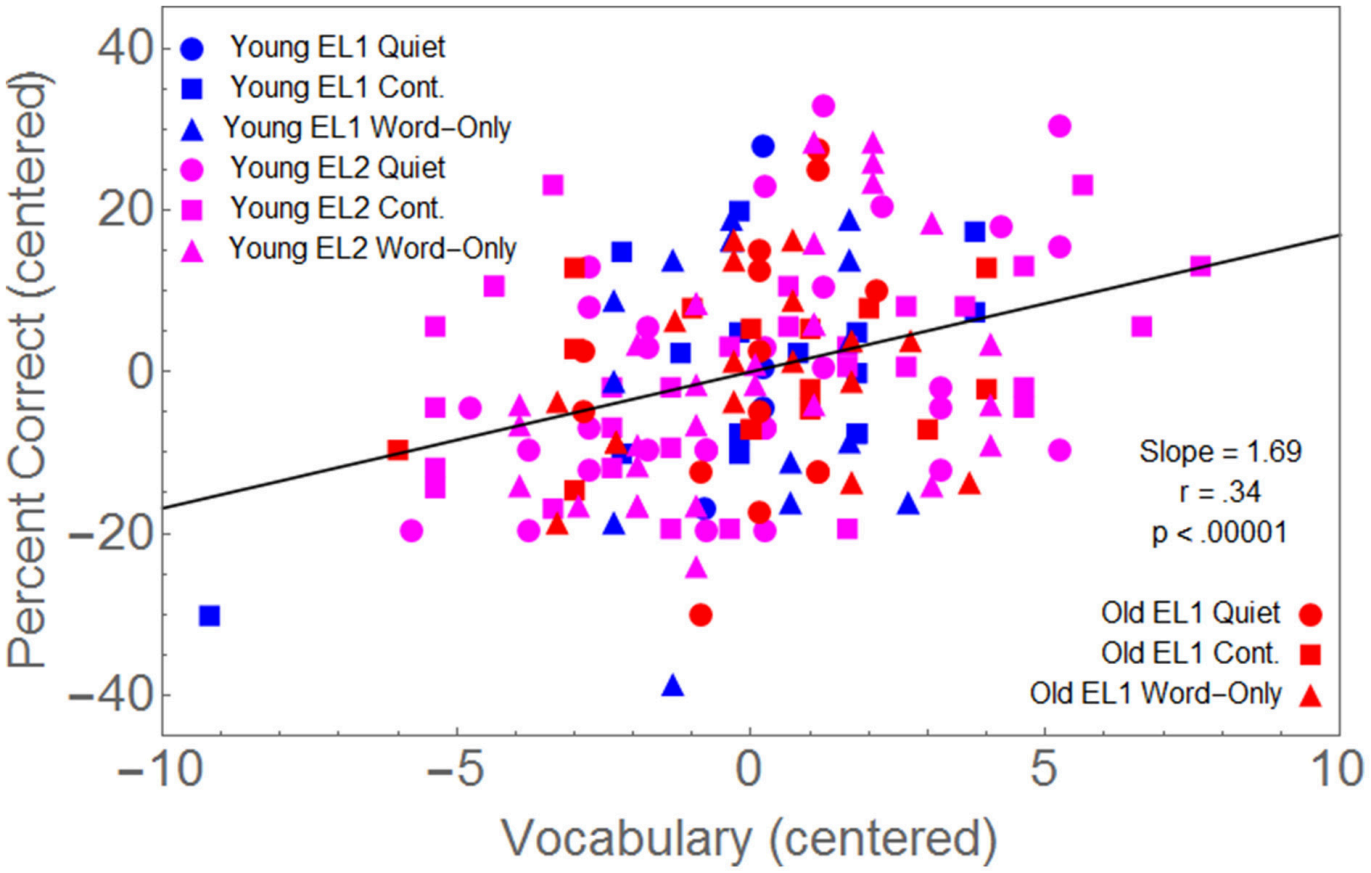
**Percent correct word recall, centered in each combination of three Groups (Y-EL1, Y-EL2, O-EL1) × 3 Masker Conditions (Quiet, Continuous Babble, Word-Only Babble), is plotted as a function of the vocabulary scores also centered in each of these nine conditions**.

Because we also had Mill Hill vocabulary scores for all but 11 of the younger and older EL1 listeners, we centered these scores, and combined them with the data from the EL2 listeners. Hence we could examine the relationship between the Mill Hill vocabulary scores (centered in each of the 3 Group × 3 Masker Condition) and the average percentage correct word recall (again centered in each of the nine groups). We first fit a model in which separate slopes were fit to each of these nine sets of data. This nine parameter model accounted for 15.2% of the data. We then compared this to a model in which a single slope was fit to all the data. Reducing the number of fitted parameters from 9 to 1 did not significantly improve the fit [*F*_(8, 162)_ < 1]. Hence, a single slope provides as good a fit to the data as a 9-slope model. Figure 5 shows that the correlation coefficient between centered percent correct and the centered Mill Hill vocabulary scores provides a good fit to the data for all combinations of Age Group × Masker Type Conditions.

Figure 5 indicates memory performance is positively related to vocabulary knowledge to the same extent in each of these three groups, and that this relationship is unaffected by the type of masker once word recognition ability has been equated in all three groups. Hence, those with greater vocabulary knowledge outperform those with lesser vocabulary knowledge, independent of their age or language status.

## Discussion

### Perceptual and cognitive measures

Because the hearing levels of the young EL1 and EL2 listeners were equivalent (both groups had thresholds within the normal range see Figure 2), we would expect both groups to be equally adept at detecting the presence of a babble of voices. However, we would expect older adults to have higher detection thresholds for speech than younger adults because of age-related hearing losses which are especially prominent in the high-frequency range (Schneider and Pichora-Fuller, 2000 for review). Consistent with this expectation, babble detection thresholds did not differ between young EL1 and EL2 listeners, with babble thresholds in both younger groups being lower than in the older EL1 listeners.

Differences in word recognition thresholds between younger and older EL1 listeners are most likely related to age-related changes in hearing. A number of studies have shown that word recognition in noisy situations is poorer in older than in younger adults when individuals from both groups are listening to speech in their native language (Dubno et al., 1984; Humes and Christopherson, 1991; Benichov et al., 2012). Age-related changes in peripheral hearing would lead to a reduction in the salience of the acoustic cues that would facilitate lexical access (Schneider et al., 2010; Rönnberg et al., 2013). Hence under equivalently noisy listening conditions we would expect older adults to recognize fewer words than younger adults.

Word recognition has been found to be poorer in young people when they are listening to speech in their second language under noisy conditions (e.g., Bradlow and Pisoni, 1999; Ezzatian et al., 2010). Here, the reasons for needing a more favorable SNR are unlikely to be due to an impoverished acoustic signal but rather to an inadequate command of the phonology, semantics, and syntax of their second language (Gollan and Kroll, 2001; Bialystok et al., 2009; Zhang et al., 2014). Moreover, when English is a person's second language, it is possible that an English word might elicit activity in both the L2 and L1 lexicons, leading to some degree of confusion (Kroll and Steward, 1994). Hence, in the absence of sufficient context, we might expect to find large differences in word recognition thresholds between young EL1 and EL2 listeners, with the extent of the difference in word recognition being dependent on the degree of exposure to and immersion in the second language (Mayo et al., 1997; Ezzatian et al., 2010).

In previous experiments (Ezzatian et al., 2010; Avivi-Reich et al., 2014, 2015) and in the present experiments, group differences in Mill Hill vocabulary scores were also observed. Specifically, the older EL1 listeners have the highest vocabulary scores, followed by the young EL1 listeners who, in turn, had significantly higher scores than the younger EL2 listeners. The latter result is not surprising given that EL1 listeners have had considerably more experience in reading and listening to material in English than those for whom English is a second language. Note, however, that older EL1 listeners have significantly higher vocabulary scores than younger EL1 listeners, a consistent finding in studies from our laboratory over the past few decades (Ben-David et al., 2015). The greater degree of vocabulary knowledge in older than in younger EL1 listeners probably reflects a lifetime of exposure to English language materials.

### The effects of linguistic competence on memory

A somewhat surprising result is that, once all individuals were equated for word recognition, the effects of serial position and the type of masker were the same for young EL1 and EL2 listeners. As mentioned in the Introduction, previous studies have found that individuals operating in a second language, tend to have a smaller vocabulary than monolinguals, appear to have more difficulty finding words (more tip-of-the-tongue states), have slower response times in naming pictures, and lower accuracy in recognizing words presented in noisy conditions (Gollan and Kroll, 2001; Bialystok et al., 2009). They also appear to have a reduced ability to discriminate fine phonemic information (Heinrich et al., 2010) and make use of linguistic cues, and may experience cross-language interference due to the activation of semantic and linguistic processes in more than a one language (e.g., Kroll and Steward, 1994; Mayo et al., 1997; Bradlow and Pisoni, 1999; Meador et al., 2000; Weber and Cutler, 2004). Although the present study equated young EL1 and young EL2 listeners with respect to word recognition, it did not compensate for their poorer semantic and linguistic skills, slower lexical access, and possible cross-language interference. As Zhang et al. (2014) pointed out, the relatively poorer 50% speech recognition thresholds of EL2 listeners whose asymptotic performance in quiet is near perfect, most likely reflects their lack of proficiency in the second language. Because in the current set of experiments, individually adjusting the SNRs at which the to-be-remembered words were presented produced near-asymptotic word recognition performance in all listeners, we would expect Zhang et al.'s argument to hold and the word recognition thresholds of EL2 listeners in this experiment to depend primarily on their proficiency in their second language (Gollan and Kroll, 2001; Bialystok et al., 2009; Zhang et al., 2014). The fact that episodic memory did not appear to differ with language competence when the listening situation was adjusted to produce equivalent word recognition suggest that the primary factor that makes it difficult for young EL2 listeners to recall heard words in noisy everyday listening situations is their poorer word recognition when they are tested at the same level as EL1 listeners, and not their poorer command of L2. In other words, equivalent word recognition implies equivalent memory performance in young listeners, independent of their language status.

Of particular interest is the fact that the substantially lower vocabulary scores found in EL2 listeners as compared to EL1 listeners had no apparent effect on word recall in these experiments. A number of studies have indicated that when listening is easy, bottom-up acoustic information is likely to be sufficient for word recognition (lexical access). However, when listening becomes difficult, listeners might need to draw on their vocabulary knowledge to facilitate word access (Mattys et al., 2009, 2010; Mattys and Wiget, 2011). It is quite likely that when young EL1 and young EL2 listeners are listening in the same situations (no compensation for differences in word recognition), the young EL2 listeners are more likely to be drawing on their vocabulary knowledge to facilitate lexical access than young EL1 listeners. In the current experiments, presenting the to-be-remembered words at a higher SNR in young EL2 than in young EL1 listeners may have the effect of boosting the acoustic signal to such a degree that there is little, if any, need to draw on vocabulary knowledge in both groups, and/or other top-down processes to achieve word recognition. If this is so, the greater vocabulary knowledge of the EL1 listeners may not give them as great an advantage in word recognition over EL2 listeners as it would when no adjustments in SNR are made. Hence, equating these two groups for word recognition may be expected to reduce any differences in the comprehension of heard speech in these two groups.

### The effects of age on memory

The age-related declines in memory found in older adults after compensating for age-related differences in word recognition (see Figure 4), could reflect age-related declines in phonetic, linguistic and semantic ability, or age-related declines in the ability to store, and/or retrieve, information from memory. We have seen that differences in memory performance between young EL1s and young EL2s disappear after equating for word recognition. Hence, we can safely assume that the reduced phonetic, linguistic and semantic abilities of young EL2 listeners has little, if any, effect on their ability to store word pairs for later recall once adjustments are made for word recognition. If we assume that adjustments to compensate for word recognition differences between younger and older adults also do compensate for any age-related declines in phonetic, semantic, and linguistic abilities, then the remaining age-related differences in memory performance most likely reflect age-related declines in the cognitive processes subsuming the storage and retrieval of information in memory. Hence these results support the notion that there are age-related losses in the ability to either transfer words into long-term storage and/or to retrieve the stored information. Such difficulties would explain why younger and older adults having equivalent recall of word pairs in the 4th or 5th serial positions in quiet but not of the word pairs in the more remote serial positions (see the top panel of Figure 4). Presumably, the word pairs in positions 4 and 5 are still in working memory, and therefore are available for prompted recall. Word recall in the more remote serial positions is likely to depend on memory for items in long-term storage. Recent models (Baddeley, 2000; Oberauer, 2002; Unsworth and Engle, 2007) reflect a growing consensus that working memory tasks are not solely dependent on either the long-term or short-term memory systems, but information in memory may exist in different states of accessibility (Oberauer, 2002). Only a limited number of items may be within a state of direct access (primary memory), while recently activated information remains in a passive state of readiness within the long term or secondary memory. When listening to word pairs in noise, the listening effort caused by the background babble might require the listener to switch attention away from maintaining items in primary memory. This might be especially challenging in a task such as the one used in the current study, as the number of items the listener has to remember exceeds four. Thus, at least some of the words must be retrieved from secondary memory (Unsworth and Engle, 2007). Age-related deficiencies in encoding or in retrieval from long-term or secondary storage, could explain the age-related deficit in quiet in these positions.

The results for memory in the presence of Continuous Babble or in Word-Only Babble indicate age-related decrements in all serial positions. Age-related declines in the perceptual and attentional processes required for extracting the word pairs from a babble of voices may be responsible for the uniform deficits seen in each serial position. When the babble background is continuous, the listener may have to continuously focus attention on the acoustic signal to facilitate processing of the word pair when it is presented, drawing resources away from maintaining the words in working memory where they can be rehearsed, and transferred to long-term memory. Age-related declines in such attentional resources could lead to the pattern of results shown in the middle panel in Figure 4. The continued decline in performance from serial position 3 to serial position 1 is also consistent with this hypothesis. If continuous babble interferes with rehearsal and transfer into long-term storage, we might expect that the more remote the word pair is from time of testing the less likely it will be recalled correctly. Hence the need to maintain focused attention on the auditory input when the babble is continuous could be the reason for the Serial Position × Masker Type interaction that is present in both younger and older adults.

The age-related decline in performance at all serial positions when the background babble begins at the same time as the word pairs is most likely due to a greater degree of sluggishness in stream segregation in older compared to younger adults. Ben-David et al. (2012) have shown that near simultaneous onset of the babble background and the word to be recognized is more deleterious to word recognition in older than in younger adults. Recall that word recognition in the two age groups is equated for individual words presented in a continuous background babble. Hence equating word recognition in a continuous babble may not produce equal word recognition when there is near simultaneous onset of the masker and the word pairs. Poorer word recognition when the onset of the babble is simultaneous with word pair onset would be expected to produce poorer memory for all serial positions in older adults. For further discussion of the effects of age on memory for paired associates please see Heinrich and Schneider (2011).

### The effects of vocabulary and reading comprehension on memory

Vocabulary size but not reading span or years of schooling contributed to individual differences in episodic memory of unrelated word pairs in EL2 listeners. Moreover, this relationship between vocabulary and memory was qualitatively the same for young EL2 listeners as for EL1 listeners of both ages. This suggests that in this particular memory task, all three groups of listeners rely on vocabulary knowledge to the same extent once perceptual differences were equated for. This result is in contrast to more conversational listening situations as will be discussed below.

### The role of memory in the comprehension of spoken language

The present results indicate that age-related declines in episodic memory persist even when steps are taken to equate all listeners with respect to their ability to recognize words in the absence of supportive context. Moreover, the failure to find episodic memory deficits in young EL2 listeners indicates that once young listeners are equated for word recognition, their degree of linguistic competence does not appear to have a major impact on their performance in this paired-associate memory task. Because the syntactic and semantic systems are relatively well-preserved in older EL1 listeners, it is unlikely that age-related changes in linguistic abilities are the source of age-related memory declines. We have suggested that these age-related deficits are related to age-related changes in perception (e.g., sluggish stream segregation), and to age-related changes in the availability or deployment of the attentional resources that are used to support episodic memory.

That age-related losses in memory persist even when the acoustic scene is adjusted for differences in word recognition in noise poses a problem for studies investigating the ability of younger and older adults to comprehend connected discourse of the kind that occurs when listening to lectures or to multi-talker conversations. Digesting the content of a lecture or following a multi-talker conversation when noise is present in the background is a complex and difficult task for any listener. For instance, in a multi-talker conversation the listener has to perceptually segregate the target speech from the background, extract the meaning of each utterance, switch attention from one talker to another, keep track of what was said by whom, store this information in memory for future use, integrate incoming information with what each conversational participant has said or done in the past, and draw on the listener's own knowledge of the conversation's topic to extract general themes and ideas (Murphy et al., 2006; Schneider et al., 2010). Higher word recognition thresholds in young EL2 and older EL1 listeners would place them at an immediate disadvantage relative to young EL1 listeners, and, indeed, their ability to answer questions about what they just heard is compromised in such a condition (e.g., Schneider et al., 2000). This raises the question of what we might expect to find in a lecture-type experiment in which listeners are required to answer questions when we equate individuals in all three groups with respect to their ability to recognize individual words in the absence of context using the same procedure that we followed in the paired-associate memory experiments described above.

Clearly, answering questions about a lecture or conversation that you have just heard has a significant memory component. The paired-associate memory experiments described above indicate that memory in younger adults appears to be independent of the language competency of the individuals as long as SNRs are adjusted to produce equivalent word recognition in all individuals. Hence one might expect comprehension differences between young EL1 and young EL2 listeners to be minimal once the listening situation is adjusted to produce equivalent word recognition. Indeed, when young EL2 and EL1 adults are asked to answer questions after listening to two- and three-person conversations, the two groups do not differ with respect to the number of questions they can answer correctly (Avivi-Reich et al., 2014, 2015) when they are equated for word recognition. But we have seen that age-related memory deficits remain after adjustments have been made to word recognition in older adults. Hence we might expect that their ability to answer questions concerning the heard material would be compromised by their poorer episodic memory even after adjusting for word recognition. The results of such experiments, however, indicate that once younger and older adult have been equated for word recognition, they can answer approximately the same number of questions correctly (Schneider et al., 2000; Murphy et al., 2006; Avivi-Reich et al., 2014, 2015). Such results indicate that older adults are able to compensate in some fashion for their poorer memory when asked to comprehend connected discourse of various kinds as long as they can hear the individual words as well as younger adults. The question then becomes how they are able to maintain good comprehension in the face of memory deficits?

There appear to be two possible explanations of how such compensation might be accomplished. The first is that there is evidence that older adults, including those with hearing loss, make better use of context when it is available. It is important to keep in mind that most episodic memory tasks are conducted with word list type material, which consists of single unrelated words. Discourse, on the other hand, contains ample context that could help in encoding and recalling information. The advantageous effect of context for older adults' memory is well known within the cognitive literature (Koutstaal and Schacter, 1997). Moreover, context not only plays an important role in memory encoding in older listeners, but also for perception. It has been previously found that older adults benefit more than younger adults from context when asked to repeat a sentence they just heard or read (Pichora-Fuller et al., 1995; Speranza et al., 2000). The SNR adjustment procedure used in the experiments where listeners were asked questions about lectures or conversations (Schneider et al., 2000; Murphy et al., 2006; Avivi-Reich et al., 2014, 2015) equated individuals with respect to their ability to recognize words in the absence of contextual support. If, after such an adjustment, older adults can make better use of context to support word recognition than can younger adults, we would expect them to actually have better word recognition than younger adults when listening to lectures or conversations. Hence the presence of context in such listening situations may compensate for older adults' poorer episodic memory for unrelated words.

Older adults are also likely to have acquired a broader world knowledge than have younger adults, which may help them to compensate for memory difficulties in conversations. World knowledge is often referred to as crystalized intelligence. Crystalized intelligence is accumulated through education and life experience, and does not appear to decline, and may even improve with age (McArdle et al., 2002). The greater one's knowledge of a culture's language history is, the more likely one is to be able to comprehend and remember discourse related to that specific culture. If older adults' crystalized intelligence is more fully developed than that of younger adults, the easier it will be for them to comprehend and remember lectures and/or conversations that are embedded in that culture^2^. Hence, a more comprehensive knowledge of the culture from which the materials were drawn in older adults could also compensate for their age-related deficits with respect to episodic memory.

A person's vocabulary knowledge is often used as a measure of one's crystalized intelligence. It has been shown consistently that older adults' knowledge of the English vocabulary has exceeded that of younger adults (Ben-David et al., 2015)^3^. Since vocabulary knowledge is often taken as a measure of crystalized intelligence, the higher vocabulary scores of older adults gives credence to the notion that their crystalized intelligence exceeds that of younger adults. Moreover, when listening to lectures and stories becomes difficult, individual differences in vocabulary scores are more predictive of individual differences in comprehension in older EL1 listeners than they are in younger EL1 or EL2 listeners (Schneider et al., 2016). It may be that under difficult listening situations older adults rely more on crystalized intelligence than do younger adults. Hence, the available evidence suggests that younger and older adults rely on different sets of abilities to achieve comparable levels of comprehension when all individuals have been equated for word recognition in the absence of context, and that their generally greater degree of world knowledge, and the greater benefit they gain from context may compensate for their poorer episodic memory for unrelated words.

## Author contributions

BS, MA, and AH designed the studies and participated in the analysis of the data. CL and MA conducted the EL2 study. BS contributed most to the writing of the manuscript with input from AH and MA. BS and AH contributed most to the revision of the manuscript and all authors approved its final version.

### Conflict of interest statement

The authors declare that the research was conducted in the absence of any commercial or financial relationships that could be construed as a potential conflict of interest.

## Footnotes

^1^Nelson Denny reading comprehension measure was not collected in the previous studies on younger and older EL1 listeners.

^2^The lectures and conversations used in (Schneider et al., 2000; Murphy et al., 2006), and (Avivi-Reich et al., 2014, 2015), were all drawn from within a North American English speaking context.

^3^A recent study (Hartshorne and Germine (2015) suggests that vocabulary knowledge reaches its peak somewhere between 55 and 70 years of age and declines thereafter (see their Figure 3). However, the average vocabulary knowledge of the older adults between the ages of 65–80 still exceeds those of 20 year olds.
